# Tools for the cryo-EM gold rush: going from the cryo-EM map to the atomistic model

**DOI:** 10.1042/BSR20170072

**Published:** 2017-12-05

**Authors:** Doo Nam Kim, Karissa Y. Sanbonmatsu

**Affiliations:** 1 Theoretical Biology and Biophysics Group, Los Alamos National Laboratory, Los Alamos, U.S.A.; 2 New Mexico Consortium, Los Alamos, U.S.A.

**Keywords:** cryo-EM, cryo electron microscopy, modeling, structural biology

## Abstract

As cryo-electron microscopy (cryo-EM) enters mainstream structural biology, the demand for fitting methods is high. Here, we review existing flexible fitting methods for cryo-EM. We discuss their importance, potential concerns and assessment strategies. We aim to give readers concrete descriptions of cryo-EM flexible fitting methods with corresponding examples.

## Introduction

In the past two years alone, more than 300 cryo-electron microscopy (cryo-EM) reconstructions with resolutions <4 Å were deposited in the Electron Microscopy Data Bank (EMDB) [1], enabling atomistic structures for the *Bacillus anthracis* pore complex, many types of viruses, membrane proteins, transcription complexes, and other macromolecular complexes. cryo-EM structures of large complexes now rival X-ray crystallography in resolution. One example of this is the 1.8 Å resolution structure of glutamate dehydrogenase by cryo-EM, whereas the highest X-ray structure resolution is 2.7 Å [1,2].

Among the four methods of cryo-EM (cryo-tomography, single particle analysis, electron crystallography, and micro-electron diffraction), single particle analysis (SPA) and cryo-tomography do not require crystallization [3]. SPA is one of the major cryo-EM approaches (78% among all cryo-EM methods) [4]. It can elucidate compositional/conformational heterogeneity and capture more native-like states of macromolecular complexes than X-ray crystallography [5]. For example, isocitrate dehydrogenase was believed as an asymmetric dimer as seen by X-ray crystallography. However, cryo-EM had shown that the apparent asymmetry is likely due to crystal lattice formation rather than intrinsic properties. Indeed, inspection of lattice packing confirmed this [6]. Other examples include medium resolution cryo-EM structures of the AMPA glutamate receptors that showed how stabilizing mutations for crystallization perturbed the native structure [7,8]. In addition, cryo-EM structures have been solved for helical polymers that cannot be crystallized in their native form because of symmetry mismatch with any crystal space group. Many proteins in cells and viruses are helical polymers [7,9]. Another example is the 2.3 Å resolution cryo-EM structure of human p97, an AAA ATPase for which crystal structures of the full-length complex have been difficult to obtain. Crystal structures of individual domains are not useful for analysis of inhibitors that bind to interfaces between these domains [7,10]. Overall, there are limitations of side chain rotamers in high resolution of X-ray crystallography [1].

## General aspects of cryo-EM

In cryo-EM SPA, larger complexes are typically easier to solve than smaller systems since the larger particles are more easily discernible with a higher signal-to-noise ratio [11]. Recently, however, cryo-EM SPA structures have been solved for proteins as small as 64 kDa [5,12] and 93 kDa (794 amino acids, PDB accession code: 5K10) [6]. A new optical device, the volta-phase plate, has been used for smaller molecules by improving contrast by an order of magnitude [13]. Since SPA can determine complexes as big as 11,200 kDa, the method is applicable to a wide distribution of sizes. Additionally, it is expected that cryo-EM SPA will be eventually applicable to determine biological structures *in vivo* [14]. cryo-EM already has been used to determine structures *in situ* either by SPA [15,16] or tomography [17,18]*.* To meet these growing needs, many cryo-EM facilities are being established around the world including United States, Europe [19], and China [20] with the latest microscopes including the *Krios* and *Arctica* [21].

However, a well-known challenge exists: the interaction with the air–water interface can damage particles when the sample becomes too thin after blotting [22]. To easily predict this status, it was recently found that streptavidin monolayer crystals can be used as an internal standard to assess single-particle cryo-EM image quality even when Thon ring qualities are the same [23]. Additionally, the spraying–plunging method using microfluidics is poised to be more readily adopted to produce highly consistent thicknesses, replacing pipetting–blotting–plunging method [24]. These endeavors for better image quality are expected to further fuel the rapid growth of cryo-EM along with newly built cryo-EM facilities.

Both map reconstruction and model building require significant compute resources. For example, it is estimated that more than half a million CPU hours of classification and high-resolution refinement were spent to generate 3.7 Å resolution structure of a spliceosomal complex [25,26]. As a result, many graphics processing unit (GPU) based applications have been and are in development [25,27–29]. It is also notable that *Nvidia*’s GPU (Tesla V100) achieves 15 teraflops for single-precision calculations [30] and the upcoming Intel memory, *Optane* is expected to reduce a realistic bottleneck of modern computers by boosting data input and output speed drastically [31]. In order to save compute time, cryo-EM modelers are advised to know that single precision calculations have shown similar accuracy as with double-precision for the widely used map reconstruction software package, *relion* [25].

A new map reconstruction software package, *cryoSPARC*, refines a 3D map almost nine times faster than the widely used GPU enabled *relion* by removing redundant computation [27]. This significant speed-up is very useful because many different approaches need to be explored to process different datasets [32]. Among many 3D classification methods, *cryoSPARC* and *relion* (starting in version 2.1) stand out, as these do not require a template for map reconstruction. Most other existing techniques for 3D classification in cryo-EM map reconstruction need a single initial reference from which discovery of heterogeneity proceeds. This unique advantage of *de novo* map reconstruction has already enabled *cryoSPARC* to find more unexpected conformations [27,33]. Finding more conformations of biomolecules opens a new horizon for flexible fitting, as it can sample more biologically relevant conformations which are distinguished by cryo-EM SPA [34]. Once time-resolved cryo-EM [24,35] matures, flexible fitting to study short-lived intermediates is expected to increase its importance. Finally, as reviewed previously [11], energy filters to decrease noise, gold films, and films made of graphene/graphene oxide to reduce motion are promising new directions.

## Toward higher resolution cryo-EM maps

Until recently, cryo-EM has been limited to intermediate resolution (5–20 Å) [36]. At these resolutions, highly accurate atomic modeling directly from the data has not been feasible and fitting methods have to be used. Here, the electron density does not have sufficiently distinctive features for an unambiguous placement of a component. One of the earliest fitting methods for low resolution cryo-EM data was core-weighting and grid-threading Monte Carlo (GTMC) [37]. The core-weighting simplifies a prohibitively large exhaustive conformational search with many components into single component fitting procedure. Then each single component fitting is performed using a GTMC that identifies the global maximum state (best fit) among a series of local maximum states determined by short Monte Carlo searches originating at a variety of grid points. Cross-linking techniques to determine the spatial distribution of proteins were also used to supplement this low-resolution map [38]. However, fitting results into low-resolution maps often had to be corrected once higher resolution maps were provided [38].

Although the resolutions of the map were not high, useful models could be obtained by fitting into a map [39]. For example, a fitted model in a low resolution map was used to predict the change of binding energy [40]. Additionally, modeling low resolution cryo-EM maps was possible using individual components from other high-resolution structure determination methods, such as X-ray crystallography, NMR, or comparative modeling [34,39,41,42]. There are several excellent previous reviews with respect to various computational fitting methods for these low-resolution maps [34,39,43].

Recent breakthroughs such as emergence of the direct electron detector camera, better motion correction, and improved contrast transfer functions [28,44] for SPA have produced cryo-EM maps with significantly higher resolutions, such as 1.8 [6] and 2.2 Å [45]. This improvement in resolution allows more in-depth analysis. For example, with 3.6 Å resolution, it was possible to see how an inhibitor interacted specifically with the *Plasmodium falciparum* proteasome, not the human one [7,46]. With respect to placing small molecules accurately, 2.5–3.0 Å resolution is typically required for X-ray crystallography derived maps: a similar effective resolution is expected for cryo-EM structures. One of the greatest examples that will prove this resolution significance is study of autophagy [22] which was discovered by transmission electron microscopy (TEM) by more than 50 years ago [47] (most modern cryo-EM uses TEM as well).

This resolution revolution has introduced different design considerations for flexible fitting. The maps tend to contain more localized noise features, making overfitting a greater challenge. On the other hand, modelers can better focus on missing maps or highly dynamic regions: simulation of cryo-EM maps with missing densities is more difficult to interpret as dynamics of biomolecules will be affected by removing domains during the fitting [48]. As resolution becomes better, molecular dynamics (MD) simulation can more effectively search conformational space, as root mean squared fluctuation (RMSF) values and local resolution are highly correlated (*r*^2^ = 0.64–0.97) [49]. Additionally, a better a consensus among different flexible fitting methods is achieved with higher resolution maps as most fitting methods are more accurate at higher resolution [39,48,50]. Even with these high resolution cryo-EM maps, using individual components from other high resolution structure determination methods is still a dominant approach [51–53].

## What is flexible fitting?

Because cryo-EM experiments can be prepared more readily and often contain many distinct conformations within a single experiment than X-ray crystallography, large numbers of cryo-EM maps are being produced by single cryo-EM labs. A bottleneck exists in going from the cryo-EM maps to the final product, namely the PDB structure.

**Figure 1:**
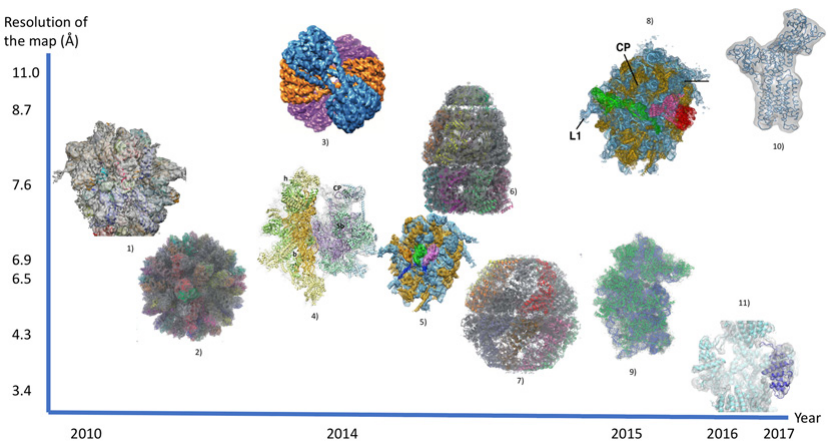
Reconstruction of biomolecules from cryo-EM maps using flexible fitting (scales of axes are arbitrary) 1) Head swivel motion on the ribosome was discovered [67]. 2) Rabbit hemorrhagic disease virus capsid (PDB 3J1P) [73]. 3) 30-nm chromatin fibers reconstituted on 12 × 187 bp DNA [72]. 4) Small subunit of the mammalian mitochondrial ribosome [64]. 5) Subunit rolling of a eukaryotic-specific ribosome was discovered [63]. 6) GroEL–GroES complex in the ATP-bound state (PDB 2C7D) [73]. 7) Mm-cpn in the closed state (PDB 3LOS) [73]. 8) Ribosomal 80S complex with termination factors [69]. 9) Entire small subunit of human ribosome fitted by cryoFIT [74]. The resolution was estimated based on article context. 10) Open structure of Ca^2+^ ATPase [57]. The resolution was estimated based on article context. 11) Transient receptor potential cation channel subfamily V member 1 (TRPV1) [49].

All structural models derived from EM maps introduce information beyond the nominal resolution [43]. Therefore, structural modeling should be performed cautiously with deep understanding of map reconstruction. Consequently, there are many backlogs of EM models compared with the number of reconstructed EM maps [34]. In order to solve this imperative need, many modeling methods for cryo-EM maps have been developed, such as rigid domain fitting (RDF), multibody fitting, flexible fitting, homology modeling, high-resolution modeling, and *de novo* modeling [54,55]: pathwalker, one of the powerful methods for *de novo* modeling, automatically traces a protein backbone from a near-atomic resolution (3–6 Å) density map and has many features such as iterative secondary structure identification, termini detection, and modeling multiple subunits without prior segmentation [56]. Among these different modeling approaches, the flexible fitting can derive a new conformational model that conforms to cryo-EM map by changing a starting structure [57]. Flexible fitting has the advantage over rigid fitting for higher resolution maps, because the rigid fitting cannot handle structural variation in electron microscopy and electron tomography maps [58]. Therefore, more than 18 different flexible fitting methods have been developed [34,43,58]. We show several examples in Figure 1. Some of the first techniques invented were simulated annealing optimization (*YUP.SCX*) [59] and normal mode flexible fitting (*NMFF*) [60,61]. One common method of flexible fitting for cryo-EM map uses molecular dynamics simulation [62,63]. Molecular dynamics flexible fitting (*MDFF*) uses prepared structures [49,64], input parameters [65], or interactive input by users [62,66]. Iterative modular fitting requires manual intervention as well, e.g. segmentation [39]. A variant on the MD theme includes all-atom structure-based molecular dynamic flexible fitting (*MDfit*) [63,67–69]. Another method (*Direx*) uses a geometry-based conformational sampling under low resolution restraints [70,71], and has been used to flexibly fit linker DNA bridging the nucleosome core into the density [72]. Below, we summarize recommended procedures before and after this flexible fitting for any method of flexible fitting.

## Data processing before flexible fitting

For more accurate flexible fitting, it is recommended to sharpen the cryo-EM map before flexible fitting [49,75] using either *Phenix* [76] or *cryoSparc* [27]. Of course, oversharpening should be avoided to prevent a partial noisy map which becomes ultimately uninterpretable [77]. After sharpening, one can manually place a starting molecule rigidly into a map using *UCSF Chimera* [78], because most flexible fitting methods require a decent level of initial cross-correlation between the model and the density [79]. In one example, the radius of convergence was reported as 25 Å in root mean square deviation (RMSD) for MD simulation based flexible fitting using progressively higher resolutions [49]. On the other hand, a direct MD simulation based flexible fitting and *Rosetta* [80] have substantially better radii of convergence. For more automated rigid-body fitting, one can use the *Fit in Map* routine in *UCSF Chimera* or *Powerfit* [79].

## Refinement after flexible fitting

Once it is confirmed that the cross-correlation (CC) value has been increased through the flexible fitting, one needs to refine modeled structures for better geometric qualities such as correct rotamer placement and protein backbone dihedral angles in accordance with Ramachandran plot, just before depositing to protein data bank (PDB) [81]. For this refinement, either *phenix.real_space_refine* [82] or *Rosetta* density-guided iterative local refinement [80] is highly recommended. Both *phenix.real_space_refine* and *Rosetta* refinement produced nearly same quality of geometries, but *phenix.real_space_refine* refined almost 20,000 times faster than *Rosetta* refinement with one test case of TRPV1 [83]. However, *Rosetta* could perturb the structure more than *Phenix* by larger backbone corrections with fragment insertion.

## Advantages of flexible fitting

It is generally regarded that flexible fitting is best suited for cryo-EM maps with resolutions between ∼3 and 9 Å [43]. If the resolution is poorer than 9 Å, the required conformational sampling can be prohibitive. If the resolution is better than 4 Å, the maps begin to show individual β-strands and side chains. In this case, *de novo* modeling becomes possible [54]. This technique can produce conformational changes that cannot be adequately modeled as movements of rigid domains alone [39,84]. In flexible fitting via MD, the MD potential is biased by a weighting factor proportional to the correlation between a cryo-EM electron density map and a simulated map based on the simulation model [63]. This method excels at deforming static X-ray crystal structures to fit the low-resolution EM data [57] and is especially useful for refining highly dynamic regions such as flexible exterior regions [62], fitting with significantly large conformational change such as closed-to-open transition [48], and loop modeling (in practice, loop regions are often deleted if they cannot be fit into cryo-EM maps, but most MD based flexible fitting can fit loop regions as well). One of the most popular methods is MDFF [49]. A second MD-based method is *MDfit*, which uses a native contact based all-atom Go model. This method has the advantage of preserving stereochemistry in the original model while simultaneously producing large conformational changes highly consistent with cryo-EM maps. In addition, the method uses a simplified model, which can fit ribosomes on a single desktop computer. *MDfit* was used to model the head–swivel transition, a key step during ribosome translocation [67]. The method also modeled a new conformational change specific to eukaryotic ribosomes (subunit rolling) [63]. In summary, when the resolution is high enough to show significant structural variation between the fitting high-resolution structure (from X-ray crystallography or NMR) and the EM maps, flexible fitting should be used [58].

For this reason, some benchmark studies have shown that flexible fitting fits better to crystal structures than RDF does [48] by giving better correlations between cryo-EM maps and modeled structures [85]. For example, it was reported that a domain in a model that has been deposited in PDB was oriented incorrectly (100° compared with an X-ray structure) by RDF [50]. For high-resolution data, flexible fitting has important advantages over modular fitting by picking up conformational changes that cannot be modeled by movements of rigid domains [39]. Flexible fitting has advantages over *de novo* methods as well. For example, when the β-galactosidase structure was modeled with different methods, flexible fitting methods generated structures with a clash score of zero, while *de novo* methods modeled a structure with a clash score of 54 [49]. Flexible fitting methods though have produced more Ramachandran outliers (1.6–3.0 %) than *de novo* methods (0.2 % Ramachandran outliers). We note that the references for Ramachandran distributions come from merely 500 files [86]. Overall, *MolProbity* scores from flexible fitting were better with ∼1.1–1.4 compared with 3.1 from *de novo* methods.

These advantages of flexible fitting are understandable, given that proteins are intrinsically flexible [87] and go through significant conformational changes. For example, because there was substantial flexibility in the structure, cryo-EM analysis had to use just 10% of the particles identified in the images [1,88]. Of course, structural changes are also induced by ligand binding or pH change as observed by solution NMR, cryo-EM single particle analysis, and room temperature X-ray crystallography [33,89,90].

## Overcoming challenges in flexible fitting

There are concerns over flexible fitting since this methodology may lead to overfitting and misinterpretation of noisy density features [39]. Restraining with secondary structure information often reduces overfitting [91]. Additionally, clustering analysis is known to be effective to avoid the overfitting problem, necessitating a large number of fitting trials to generate ensembles [57]. This approach (sampling ensemble of conformations and clustering later) has been used for *Rosetta* protein structure prediction as well [92], and makes sense given the inherent protein dynamics and its corresponding cryo-EM map space as shown with RMSF values [49].

Additionally, there is a concern over stereochemistry errors from MD simulation based flexible fitting. For example, because force fields in MD simulation do not have any energy term to enforce given chirality or *cis/trans* peptide bond configuration, an external force to fit to cryo-EM map may result in artificial chirality or peptide bond configuration [62]. However, the *MDfit* and hybrid MD technique (cryoFIT) [74] help to remedy this problem since its potential is based on initial native contact and dihedral values from the starting structure.

Finally, one caveat when applying flexible fitting to high-resolution maps is that steep wells in which the structure can be trapped are commonly found. To overcome these wells, map-restrained self-guided Langevin Dynamics (*SGLD*) was applied [93]. The SGLD selectively enhances and suppresses molecular motions based on their frequency to accelerate conformational searching without modifying energy surfaces or raising temperature. The restraint maps are included in simulations as movable objects (rigid domains) that interact with simulation systems and move with their restraining atoms. Another method of surpassing these wells was applying low-pass or Gaussian filter to the cryo-EM density map smoothing the resulting potential energy function [57,62,67].

## Quality assessment of flexible fitting

**Figure 2:**
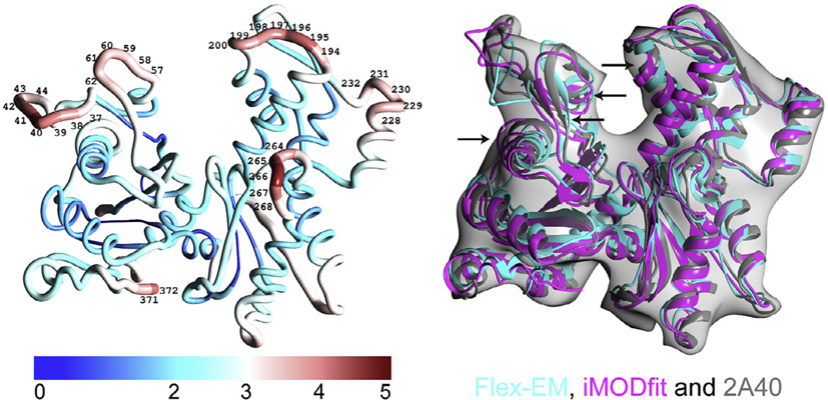
Modeling errors for the case of flexible fitting of an actin subunit homology model into simulated map. Left: Local residue error (QMEAN, which is calculated with geometrical structural descriptors in Å). Error values above 3.5 Å that are considered unreliable are labeled. Right: Comparison of flexible fits obtained using *Flex-EM, iMODfit*, and the target fit (PDB ID 2A40) [36].

It is believed that general consensus from various flexible fitting methods often is quite close to experimentally determined structures. This way of assessment is often referred as a battery approach. This argument brought a concern since all flexible fitting methods may have some systematic errors [39]. Using multiple flexible fitting offers no assurance about which model is optimal [43] as evidenced with a benchmark of 12 different proteins [50]. However, the battery approach is a well-established method of statistical assessment with respect to experiments [94–96] and prediction modeling [97]. One of the most representative examples of a battery approach not related to cryo-EM flexible fitting is a random forest which is a collection of decision trees whose outputs are summed to derive a final prediction by majority voting [98]. Indeed, when modelers validated this consensus approach with many X-ray crystallography derived structures, they found a very high correlation between consensus level and model accuracy [48,50]. For example, a correlation (*r*^2^) between the mean RMSD among the different models (a measure of consensus) and the mean RMSD between the models and the target structure for 12 different proteins was 0.99. This high correlation between consensus level and model accuracy was observed not only in global regions of the protein, but also in local regions. On top of that, at least *NMFF, MDfit, YUP.SCX*, and *DireX* are diverse enough not to have consensus on wrong solutions and yet to have consensus on correct solutions. On the other hand, the CC between the density map and the fitted model shows a very low correlation (*r*^2^ = 0.31) with RMSD from targets [50]. Therefore, the battery approach is useful to confer modeling confidence to identify local regions that are likely to fit well. A similar conclusion was made with other flexible methods as well: *flexEM* and *iMODfit* [36] (Figure 2). Not only did they derive the same conclusion, but also they improved the fit by using both of these flexible fitting methods (first they identified poorly fitted region by comparing results from two flexible fitting methods, then they improved that region only while keeping the rest of the structure fixed).

## Comparison of some flexible fitting methods

Although there are at least 18 different flexible fitting methods [34,43,58], there is no system-wide level of comparison to our knowledge. However, there are several benchmark papers. Ahmed et al. [48] tested *NMFF, MDfit*, and *YUP.SCX* with five different proteins. Among these methods, *MDfit* and *YUP.SCX* fitted structures (elongation factor 2) as close as 1.4 Å RMSD even when the required conformational changes are large (on average 9 Å). The initial structures needed to undergo a closed-to-open transition in order to fit into the EM maps. With respect to convergence, *MDfit* was most robust among the three approaches. Ahmed et al. [50] also tested *NMFF, MDfit, YUP.SCX*, and *DireX* with 12 different proteins. Overall, *MDfit, YUP.SCX*, and *DireX* showed high agreement in conformation with each other and resulted in the lowest mean RMSD from the target structure.

When *MDFF, DireX, Rosetta, Gorgon*, and *Froda* were tested with 1–7 biological systems, *MDFF* and *Rosetta* produced models free from chirality errors and *cis* peptide [99]. *MDFF* prevented stereochemical errors using harmonic restraints and *Rosetta* prevented these errors using experimentally derived force field and rotamers with fragment insertion [100].

When comparing speed between replica cryo-EM map resolution exchange *MDFF* and simple cascade of resolution change *MDFF*, replica exchange method converged 8–14 times faster than simple cascade one with similar fitting accuracy [49].

With respect to model accuracy, *Rosetta* refinement generated more accurate models than the direct *MDFF* [80]. Additionally, the accuracy of *Rosetta* refined models was independent of the starting-model quality when using cryo-EM maps of 4.5 Å or better resolution. However, when *MDFF* used cascade resolution of maps (*cMDFF, ReMDFF*), both *MDFF* and *Rosetta* generated comparable results [49].

**Figure 3:**
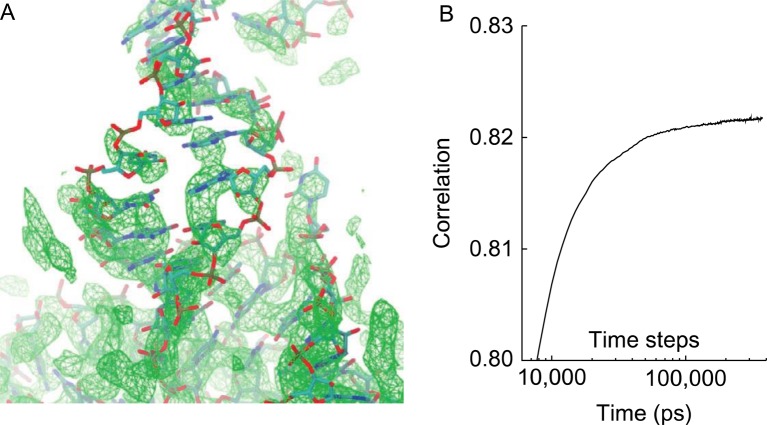
An example of cryoFIT used to fit high resolution cryo-EM map of the human ribosome [101]. (**A**) cryo-EM map of h40–h44 segment. (**B**) Time evolution of fit during simulations (courtesy Kirmizialtin et al., 2015) [74].

To specifically address higher resolution cryo-EM maps, a hybrid MD technique (cryoFIT) was developed to maintain stereochemistry of the initial starting structure while having the capability to form remodeled secondary structures and local folds [74] (Figure 3). This method combines a native contact potential with the *ab initio* potentials such as *Amber*. The method has shown promising results on the human ribosome.

## Outlook

It is expected that more MD based flexible fitting methods will adopt replica exchange or simulated annealing along with application of different Gaussian blurs of various half-widths to the experimental density as the hybrid MD simulation fitting method (cryoFIT) [74] and *MDFF* did [49], as the evidence of benefits using those enhanced sampling methods for flexible fitting has accumulated [57]. Whether the replica exchange is for either traditional temperature change or cryo-EM map density, these enhanced samplings are to overcome energetic local minima efficiently. Similarly, ensemble based sampling [57] such as RMSF [49] is also expected to be applied in order to better assess model quality. These methods also have complementary benefits to traditional CC based fit assessment.

When it comes to quantitative characterization of domain motions, minor residue mismatch often makes it challenging to compare two different structures. Moment of inertia tensors (after near-automated segmentation) may aid with this comparison [102]. This method, implemented in *VMD*, had shown its practical usefulness for ribosome with atomic structure derived maps. However, it is applicable to any molecular machines and its further development with cryo-EM derived maps is done as well.

To sample conformational space more effectively, additional constraints from evolutionary information from aligned homologous proteins are expected to be added to MD simulation based flexible fitting methods. In this way, one can find biologically more meaningful solutions by sampling more relevant conformations with reduced simulation time. The use of evolutionary conservation of residues is already being used to detect incorrectly fitted proteins [103,104] in cryo-EM maps, to predict protein structures [105,106] and druggable interfaces of protein–protein interactions [107].

To model symmetric molecules more effectively, symmetry restraints as realized in *MDFF* [108] can be considered to be applied to other flexible fitting methods as well. *MDFF* allows users to use *Amazon Web Service Cloud* platform that costs merely $0.8–1.7 for carbon monoxide dehydrogenase fitting [49,109]. Additionally, it presented GUI based applications that enable convenient model quality check plugins such as *cispeptide*, chirality, TorsionPlot, and Timeline [62].

One thing to note in this expanding field is that an increasing number of cryo-EM structures are being reported from dynamic protein assemblies [7] such as nuclear complexes that have been difficult to study conventionally [11]. Therefore, flexible fitting methods will be more important tools in this gold-rush era for cryo-EM.

## Abbreviations

CC: cross-correlation
cryo-EM: cryo-electron microscopy
GTMC: grid-threading Monte Carlo
MD: molecular dynamics
MDFF: molecular dynamics flexible fitting
NMFF: normal mode flexible fitting
RMSD: root mean square deviation
SGLD: self-guided Langevin Dynamics
SPA: single particle analysis
